# The Pathogenesis of Chronic Kidney Disease (CKD) and the Preventive and Therapeutic Effects of Natural Products

**DOI:** 10.3390/cimb47100853

**Published:** 2025-10-16

**Authors:** Yuxin Dong, Yanqing Tong

**Affiliations:** College of Traditional Chinese Medicine, Changchun University of Chinese Medicine, Changchun 130117, China; 23102570143@stu.ccucm.edu.cn

**Keywords:** CKD, pathogenesis, natural products, metabolic dysregulation, chronic inflammation, oxidative stress, endoplasmic reticulum stress, ferroptosis

## Abstract

Chronickidney disease (CKD) poses a major global public health challenge, driven by a complex pathogenesis involving multiple interconnected processes—including metabolic disturbances, chronic inflammation, oxidative stress, endoplasmic reticulum stress, and ferroptosis—which collectively contribute to progressive and often irreversible loss of renal function. Although current standard therapies can ameliorate CKD progression, a substantial number of patients still advance to end-stage renal disease, highlighting the urgent need for innovative treatment strategies. Natural products have shown great promise in the prevention and management of CKD, largely attributable to their multi-target and multi-pathway synergistic effects. This review systematically outlines the core pathogenic mechanisms underlying CKD and elucidates the molecular mechanisms through which bioactive natural compounds exert renoprotective effects. Despite robust preclinical evidence, the clinical translation of these compounds remains hindered by limitations such as poor bioavailability and a lack of large-scale clinical trials. Moving forward, research should prioritize clinical translation of these compounds, aiming to provide novel therapeutic perspectives for CKD management.

## 1. Introduction

Chronic kidney disease (CKD) is a clinical syndrome characterized by irreversible changes in kidney structure and/or function, caused by various etiologies. It has a long course, poor prognosis, and multiple complications, making it one of the urgent public health issues worldwide. With the rapid development of society and the increasing aging population, the prevalence of metabolic diseases, cardiovascular diseases, and other conditions that can lead to CKD has been rapidly increasing. According to statistics, CKD affects an estimated 850 million people globally, with a subset of patients progressing to end-stage renal disease that requires renal replacement therapy [1,2]. The pathophysiology of CKD involves a complex network of interrelated mechanisms. Recent socioeconomic development has been associated with dietary patterns characterized by excessive nutrient intake and sedentary lifestyles, which collectively contribute to metabolic dysregulation. This dysregulation promotes ectopic lipid deposition in the kidney and damages renal parenchyma [3,4]. Furthermore, chronic inflammation, oxidative stress, endoplasmic reticulum stress, and ferroptosis are closely implicated in the development and progression of CKD [5,6,7]. These interconnected mechanisms collectively drive renal fibrosis, leading to irreversible loss of kidney function.

Current therapeutic strategies, including intensive lifestyle interventions and pharmacological approaches such as renin–angiotensin–aldosterone system inhibitors (RAASis) and sodium-glucose cotransporter-2 inhibitors (SGLT2is) for managing blood pressure, glucose levels, and proteinuria, can partially slow the progression of chronic kidney disease (CKD). Nevertheless, a considerable number of patients still inevitably progress to end-stage renal disease (ESRD), ultimately becoming dependent on renal replacement therapy for survival [8,9]. Therefore, exploring other potentially effective novel treatments to ameliorate renal impairment in CKD constitutes an urgent research priority.

Within the complex pathological network of CKD, natural products demonstrate unique advantages due to their multi-target and multi-pathway synergistic effects. A systematic evaluation of clinical trials by Josa et al. confirmed that bioactive compounds such as curcumin, sulforaphane, and emodin exert nephroprotective effects in CKD patients through anti-inflammatory and antioxidant activities [10]. Furthermore, a 2024 meta-analysis indicated that Cordyceps sinensis alleviates renal inflammation and reduces plasma creatinine and urea nitrogen levels, suggesting its potential as an adjunct therapy for CKD patients undergoing renal replacement therapy [11]. These findings collectively support the unique and broad therapeutic potential of natural compounds in CKD management. This review systematically examines the key pathogenic mechanisms of CKD, details the renoprotective effects and molecular mechanisms of various natural products, and discusses current research limitations and future directions, aiming to provide novel insights into therapeutic strategies for CKD.

## 2. Methods

A comprehensive literature search was conducted using electronic databases, including PubMed, Web of Science, Google Scholar, and Embase, to identify publications from January 2000 to August 2025 related to the pathogenesis of chronic kidney disease and natural product interventions. The search strategy incorporated a combination of free-text terms and subject headings, such as “chronic kidney disease”, “renal fibrosis”, “nephropathy”, “kidney disease”, “renal failure”, “renal injury”, “pathogenesis”, “natural product”, “herb”, as well as specific compound names, such as “Curcumin” and “Resveratrol”. Inclusion criteria encompassed original research (both basic and clinical studies) and systematic reviews. Exclusion criteria consisted of case reports, conference abstracts, studies with incomplete or inaccessible data, and publications with uninterpretable outcomes. For duplicate publications, only the version with the most comprehensive data or the earliest publication date was retained. Literature screening and data extraction were performed independently by two researchers, with any discrepancies resolved through consensus discussion.

## 3. Pathogenesis

### 3.1. Metabolic Dysregulation

**Figure 1:**
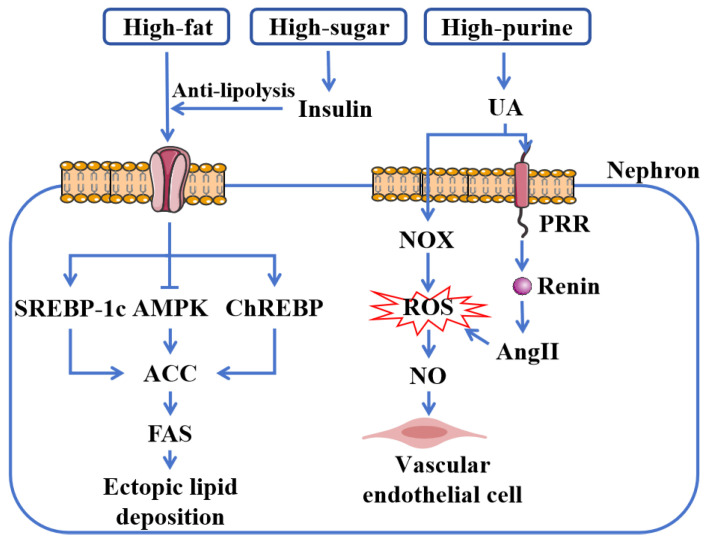
Metabolic dysregulation in CKD. A high-fat and high-sugar diet contributes to renal ectopic lipid deposition by elevating FFAs. Insulin, which exerts an anti-lipolytic effect, plays a pivotal role in this process. These fatty acids subsequently promote lipid deposition by upregulating SREBP-1c and ChREBP and downregulating AMPK, which in turn leads to the activation of ACC and the upregulation of FAS, further driving lipid synthesis. Furthermore, a high-purine diet induces renal endothelial cell injury through a mechanism mediated by AngII and NOX-derived ROS. Abbreviations: SREBP-1c, sterol regulatory element-binding protein-1c; AMPK, AMP-activated protein kinase; ChREBP, carbohydrate response element-binding protein; ACC, acetyl-CoA carboxylase; FAS, fatty acid synthase; UA, uric acid; NOX, NADPH oxidase; ROS, reactive oxygen species; NO, nitric oxide; PRR, pro-renin receptor; AngII, angiotensin II.

A genome-wide association analysis of 157 European CKD patients revealed that metabolic dysregulation is one of the primary pathways contributing to CKD progression (Figure 1) [12]. Under physiological conditions, substances such as glucose, uric acid, and lipids maintain dynamic equilibrium, and disruption of this balance—due to either overproduction or impaired excretion—constitutes a major cause of metabolic dysregulation. Prolonged consumption of a high-sugar, high-fat, or high-purine diet (where intake exceeds expenditure), as well as abnormal expression of metabolic enzymes, can lead to the accumulation of these metabolites, ultimately resulting in renal impairment.

With the growing population of obese individuals, we have observed that diet and other lifestyle factors play a crucial role in the development and progression of CKD. Prolonged high-calorie diets contribute to “ectopic lipid accumulation”—a condition in which excess lipids deposit in non-adipose tissues when energy intake surpasses adipose storage capacity [13]. Studies indicate that lipids can accumulate in virtually all renal cell types, including mesangial cells, podocytes, and proximal tubular cells [3,14]. This ectopic lipid deposition is considered “lipotoxicity” and can induce the toxic effects in cellular injury, collectively promoting renal fibrosis [15,16,17].

The upregulation of key transcriptional regulators, including sterol regulatory element-binding protein-1c (SREBP-1c) and carbohydrate response element-binding protein (ChREBP), coupled with downregulation of AMP-activated protein kinase (AMPK), promotes renal ectopic lipid deposition. This cascade subsequently activates acetyl-CoA carboxylase (ACC), leading to increased fatty acid synthase (FAS) expression and enhanced lipogenesis, thereby exacerbating renal lipid accumulation [18,19,20,21]. Animal studies have confirmed that mice deficient in AMPK activity develop lipid droplets in the proximal renal tubules [22]. Alterations in the expression or function of these molecules have also been consistently observed in CKD patients [23].

Furthermore, insulin resistance, frequently associated with CKD, exacerbates glomerular hyperfiltration, leading to glomerulosclerosis and renal dysfunction [24,25]. Additionally, insulin’s anti-lipolytic effect further promotes lipid accumulation, establishing a vicious cycle that perpetuates metabolic dysregulation [26].

Uric acid also contributes directly to renal injury. Derived mainly from dietary sources and nucleotide metabolism, uric acid is the end product of purine metabolism. As a pro-oxidant, it activates NADPH oxidase (NOX), inducing oxidative reactions and generating free radicals [27,28]. Additionally, uric acid stimulates the pro-renin receptor (PRR) in proximal tubular cells, upregulating the expression of renin and excessively activating the renin–angiotensin system (RAS). This leads to increased angiotensin II (AngII) production, elevated intracellular reactive oxygen species (ROS), inducing oxidative stress, suppression of nitric oxide (NO) release, and mediating endothelial cell injury, collectively contributing to renal dysfunction [29,30].

### 3.2. Chronic Inflammation and Oxidative Stress

**Figure 2:**
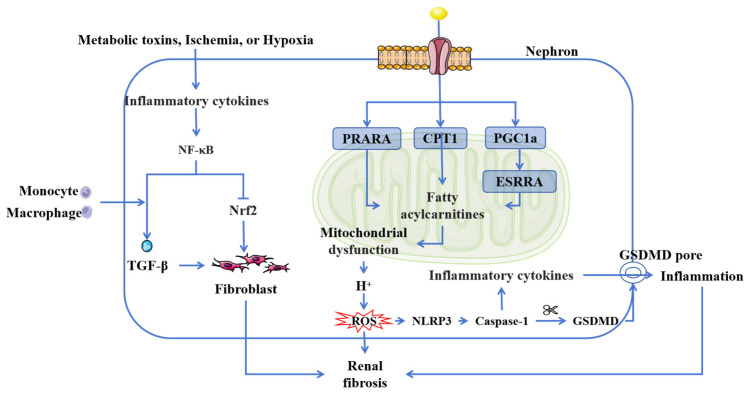
Inflammation and oxidation in CKD. Chronic inflammation and oxidative stress are related to the NF-κB inflammatory signaling pathway and mitochondrial function. NF-κB activation further exacerbates oxidative stress by suppressing Nrf2. Mitochondrial fatty acid metabolism is central to this process. The reduced expression of CPT1, coupled with suppressed PPARA and PGC1a activity, initiates a pathogenic cascade. Furthermore, the downregulation of PGC1a inhibits ESRRA expression, leading to the accumulation of fatty acylcarnitines and subsequent mitochondrial dysfunction. This dysfunction promotes excessive ROS production, which triggers the assembly and activation of the NLRP3 inflammasome. Subsequent caspase-1 activation cleaves GSDMD, forming membrane pores and ultimately inducing pyroptosis. Abbreviations: TGF-β, transforming growth factor-beta; Nrf2, Nuclear factor erythroid-2-related factor 2; NF-κB, nuclear factor kappa-B; PPARA, peroxisome proliferator-activated receptor alpha; CPT1, carnitine palmitoyltransferase-1; PGC1a, peroxisome proliferator-activated receptor gamma coactivator 1-alpha; ESRRA, estrogen-related receptor alpha; ROS, reactive oxygen species; GSDMD, gasdermin D.

Chronic inflammation is widely recognized as a pivotal driver in the initiation and progression of CKD, forming a self-reinforcing vicious cycle through its interplay with oxidative stress and mitochondrial dysfunction. Distinct from acute inflammation, chronic inflammation represents a maladaptive response triggered by persistent activation of pro-inflammatory signaling pathways. When renal resident cells are exposed to metabolic toxins, ischemia, or hypoxia, sustained inflammatory responses are activated, ultimately leading to renal interstitial fibrosis [31] (Figure 2). Hyperactivation of the key pro-inflammatory transcription factor nuclear factor kappa B (NF-κB) competitively suppresses nuclear factor erythroid 2-related factor 2 (Nrf2) and promotes immune cell infiltration [32,33]. This process upregulates pivotal pro-fibrotic factors such as transforming growth factor-beta (TGF-β), facilitating the transition of epithelial and mesangial cells into fibroblasts and myofibroblasts, which in turn stimulates collagen and extracellular matrix (ECM) protein secretion, thereby accelerating renal fibrosis.

Chronic inflammation is closely linked with oxidative stress. The kidney, particularly renal tubular cells, is rich in mitochondria to meet its high energy demands, making mitochondrial health essential for renal function [34]. Mitochondria primarily generate energy through fatty acid oxidation (FAO) and oxidative phosphorylation (OXPHOS). However, mitochondrial dysfunction is prevalent in CKD progression [35]. Studies of CKD have revealed significant downregulation of genes associated with FAO and OXPHOS [36,37]. Key manifestations include reduced expression of carnitine palmitoyltransferase-1 (CPT1), a rate-limiting enzyme in mitochondrial FAO, along with suppressed activity of peroxisome proliferator-activated receptor alpha (PPARA) and peroxisome proliferator-activated receptor gamma coactivator 1-alpha (PGC1a) [38,39,40]. Downregulated PGC1a further inhibits estrogen-related receptor alpha (ESRRA) expression, impairing fatty acid oxidation and resulting in the accumulation of medium-chain and short-chain acylcarnitines [41]. Additionally, PGC1a and ESRRA serve as critical regulators of mitochondrial biogenesis and function, reflecting mitochondrial dysfunction [42].

Mitochondrial dysfunction disrupts the proton gradient across the inner mitochondrial membrane, leading to hydrogen ion leakage that reacts with oxygen and triggers a chain reaction of reactive oxygen species (ROS) generation [43,44]. As a key mediator of kidney injury, ROS activates the NLRP3 inflammasome, promotes caspase-1-mediated cleavage of gasdermin D (GSDMD), and induces pyroptosis—a form of programmed cell death characterized by pore formation in the cell membrane. This process facilitates the release of cellular contents and pro-inflammatory cytokines such as interleukin-1β (IL-1β) and interleukin-18 (IL-18), thereby amplifying inflammatory responses and accelerating CKD progression [45,46]. In summary, chronic inflammation, oxidative stress, and mitochondrial dysfunction are causally interrelated and collectively drive the pathogenesis and progression of CKD.

### 3.3. Endoplasmic Reticulum Stress

**Figure 3:**
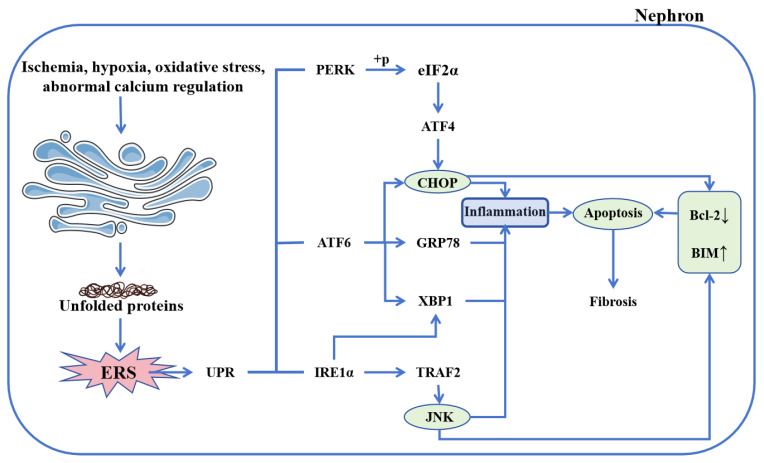
Endoplasmic reticulum stress in CKD. The imbalance of endoplasmic reticulum homeostasis triggered by various factors initiates UPR, which is mediated by three key sensors: PERK, ATF6, and IRE1α. PERK activation phosphorylates eIF2α, leading to ATF4 upregulation and subsequent induction of CHOP expression. ATF6 enhances both CHOP and GRP78 expression, while also acting synergistically with IRE1α to promote XBP1 activation. Furthermore, IRE1α recruits TRAF2 to activate JNK, thereby initiating inflammatory signaling and promoting apoptosis. Both activated CHOP and JNK converge to suppress Bcl-2 while stimulating BIM, collectively driving apoptosis. Abbreviations: ERS, endoplasmic reticulum stress; UPR, unfolded protein response; PERK, protein kinase RNA-like endoplasmic reticulum kinase; ATF6, activating transcription factor 6; IRE1α, inositol-requiring enzyme 1 alpha; ATF4, activating transcription factor 4; CHOP, CCAAT/enhancer-binding protein homologous protein; GRP78, glucose-regulated protein 78; XBP1, X-box binding protein 1; TRAF2, tumor necrosis factor receptor-associated factor 2; JNK, c-Jun N-terminal kinase; Bcl-2, B-cell lymphoma-2; BIM, Bcl-2 interacting mediator of cell death.

Endoplasmic reticulum stress (ERS) represents a significant pathological mechanism in the progression of CKD. Under physiological conditions, the endoplasmic reticulum serves as an intricate membrane-bound organelle responsible for maintaining protein homeostasis—including synthesis, folding, processing, and trafficking—as well as regulating lipid synthesis and distribution [47,48]. However, during CKD progression, endogenous and exogenous factors such as tissue ischemia, hypoxia, oxidative stress, environmental toxins, and medications disrupt ER homeostasis, leading to sustained ERS (Figure 3). Persistent ERS leads to the continuous accumulation of misfolded or unfolded proteins, which, beyond a critical threshold, activate the unfolded protein response (UPR) and subsequently contribute to renal injury [6,49].

The UPR is mediated through three primary signaling branches involving protein kinase RNA-like endoplasmic reticulum kinase (PERK), inositol-requiring enzyme 1 alpha (IRE1α), and activating transcription factor 6 (ATF6), which collectively function as sensors of ER stress [50]. Sustained ERS promotes PERK activation, resulting in phosphorylation of eukaryotic initiation factor 2α (eIF2α), upregulation of ATF4 expression, and induction of C/EBP homologous protein (CHOP) [51,52,53]. Concurrently, unmitigated ERS enhances IRE1α expression, leading to activation of X-box binding protein 1 (XBP1) and recruitment of TNF receptor-associated factor 2 (TRAF2), activating c-Jun N-terminal kinase (JNK), thereby initiating inflammatory signaling [54,55]. ATF6, a type II ER transmembrane protein, upregulates the expression of glucose-regulated protein 78 (GRP78), XBP1, and CHOP under persistent ERS conditions [54,56]. Activated CHOP and JNK further suppress the anti-apoptotic protein B-cell lymphoma-2 (Bcl-2) while promoting expression of the pro-apoptotic Bcl-2 interacting mediator of cell death (BIM), collectively driving apoptosis [57,58,59].

Furthermore, ERS is closely associated with other CKD pathogenic mechanisms, including oxidative stress and inflammation. The accumulation of misfolded proteins in the ER enhances reactive oxygen species (ROS) generation and depletes glutathione (GSH), ultimately inducing oxidative stress [60,61]. Interestingly, GRP78, an ER molecular chaperone, not only facilitates protein folding and calcium binding but also modulates innate and adaptive immune responses, thereby inducing an inflammatory response [62]. Chronic ER stress also leads to overactivation of XBP1s and CHOP, which exacerbate inflammatory signaling, induce proliferation and deposition of extracellular matrix, accelerating the progression of renal fibrosis [63,64]. These findings collectively indicate that ERS contributes to the progression of CKD to end-stage renal disease (ESRD) and highlight its potential as a therapeutic target in chronic kidney disease.

### 3.4. Ferroptosis

In the early 21st century, Dolma et al. identified a novel compound named erastin, which selectively kills cancer cells through a non-apoptotic mechanism that can be significantly inhibited by iron chelators [65,66]. In 2012, Dixon termed this iron-dependent form of cell death “ferroptosis” [67]. The core mechanism involves intracellular iron accumulation, which triggers lipid peroxidation of polyunsaturated fatty acids in cell membranes, leading to toxic lipid peroxide buildup, elevated reactive oxygen species (ROS), and subsequent cell death [68]. Recent studies indicate that ferroptosis is co-regulated by multiple metabolic pathways, including iron metabolism, lipid metabolism, amino acid metabolism, redox homeostasis, and mitochondrial activity [69,70,71,72].

**Figure 4:**
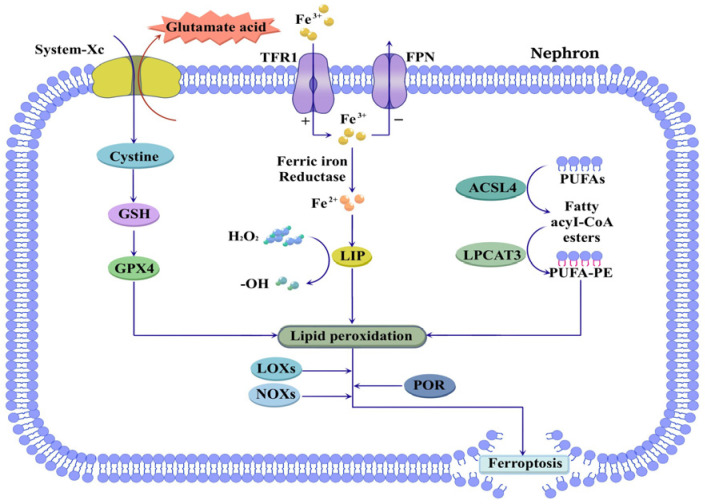
Ferroptosis in CKD. Ferroptosis is closely associated with dysregulation of iron and lipid metabolism. Increased TFR1 activity and decreased FPN expression contribute to intracellular iron overload, resulting in the formation of LIP that catalyzes free radical generation. Concurrently, GSH depletion via System-Xc inhibition suppresses GPX4 activity. Furthermore, ACSL4 and LPCAT3 mediate the esterification of PUFAs into PUFA-PE, and further promote ferroptosis through LOXs, NOXs, and POR. Abbreviations: TFR1, transferrin receptor 1; FPN, ferroportin; LIP, labile iron pool; GSH, glutathione; GPX4, glutathione peroxidase 4; ACSL4, acyl-CoA synthetase long chain family member 4; LPCAT3, lysophosphatidylcholine acyltransferase 3; PUFA, polyunsaturated fatty acid; PUFA-PE, polyunsaturated fatty acid-phosphatidyl ethanolamine; LOX, lipoxygenase; NOX, NADPH oxidase; POR, cytochrome P450 oxidoreductase.

During the progression of CKD, dysregulations in lipid and iron metabolism are commonly present, providing a compelling rationale for investigating ferroptosis in CKD (Figure 4) [13,73]. System-Xc, composed of SLC7A11 and SLC3A2 subunits, is an amino acid antiporter that mediates the exchange of extracellular L-cystine and intracellular L-glutamate. It plays a vital role in glutathione (GSH) synthesis, a key antioxidant essential for maintaining cellular oxidative balance [74]. Inhibition of System-Xc reduces GSH levels, impairs glutathione peroxidase 4 (GPX4) expression, exacerbates lipid peroxide accumulation, and ultimately induces ferroptosis [75]. Studies have confirmed that inhibition of GPX4 in CKD promotes renal ferroptosis and accelerates kidney fibrosis progression [76,77].

Under normal physiological conditions, iron regulatory proteins maintain iron homeostasis, preventing iron deposition in the kidneys. However, in CKD patients, dysregulation of iron metabolism-related proteins leads to abnormal intracellular iron accumulation [78]. Circulating iron, primarily in the Fe^3+^ form, is taken up by cells via transferrin receptor 1 (TFR1) and exported through ferroportin (FPN). Upregulation of TFR1 and downregulation of FPN result in elevated intracellular iron content [79,80]. Intracellular iron is reduced from Fe^3+^ to Fe^2+^ by ferric reductases; excess Fe^2+^ forms an unstable labile iron pool (LIP), where it catalyzes the conversion of hydrogen peroxide into hydroxyl radicals and other ROS via the Fenton reaction. This promotes lipid peroxidation, generates substantial lipid peroxides, damages membrane systems, and ultimately induces cell death [81]. Therefore, targeting iron metabolism-related proteins has emerged as a potential therapeutic strategy for CKD. Restoring renal iron homeostasis and mitigating iron-dependent ferroptosis may delay the onset and progression of CKD.

Ectopic lipid deposition is observed in nearly all renal cell types in CKD and correlates positively with disease progression [13,82,83]. The core mechanism of ferroptosis is iron-dependent lipid peroxidation, in which polyunsaturated fatty acids (PUFAs) serve as key substrates. During ferroptosis, PUFAs are activated by two membrane-remodeling enzymes: acyl-CoA synthetase long-chain family member 4 (ACSL4) and lysophosphatidylcholine acyltransferase 3 (LPCAT3). ACSL4 catalyzes the esterification of free PUFAs to acyl-CoA esters, which are then converted to polyunsaturated fatty acid-phosphatidyl ethanolamine (PUFA-PE) by LPCAT3 [84,85,86]. PUFA-PE, as the lipid peroxidation substrate, is further oxidized by lipoxygenases (LOXs), NADPH oxidases (NOXs), and cytochrome P450 oxidoreductase (POR), generating abundant radicals that drive ferroptosis [87,88,89,90]. In summary, targeting key proteins involved in the ferroptosis pathway represents a promising therapeutic strategy for CKD patients. This approach can effectively ameliorate intracellular iron overload, inhibit the accumulation of lipid peroxidation substrate PUFA-PE, improve oxidative stress, and block the lipid peroxidation cascade, thereby ameliorating renal injury.

## 4. Treatment

In recent years, natural plant-extracted compounds have gained significant attention as promising therapeutic agents for CKD. These compounds demonstrate considerable therapeutic potential in the treatment of renal pathologies, characterized by multi-target and multi-pathway synergistic effects, and demonstrate renal protective activities through mechanisms including regulation of metabolic dysregulation, anti-inflammatory actions, antioxidant effects, amelioration of endoplasmic reticulum stress, and suppression of ferroptosis (Table 1).

### 4.1. Flavonoids

Quercetin (QR), a naturally occurring flavonoid abundant in various vegetables and fruits, possesses multiple pharmacological properties, including anti-lipid deposition, anti-inflammatory, and antioxidant activities. Studies indicate that quercetin activates AMPK mRNA expression, upregulates PPARA and CPT1, and suppresses SREBP-1 expression, thereby reducing triglyceride levels in renal tubular epithelial cells, ameliorating lipid deposition, and decreasing urinary albumin (ALB) and β2-microglobulin (β2-MG) excretion, effectively attenuating lipid deposition-induced renal injury [91]. Furthermore, in vitro experiments demonstrate that quercetin inhibits NF-κB activation and reduces expression of tumor necrosis factor-alpha (TNF-α) and TGF-β1, significantly alleviating renal damage mediated by chronic inflammation [92]. Quercetin also exhibits protective effects against endoplasmic reticulum stress (ERS) by activating Sirtuin-1 (SIRT1), suppressing acetylation of eIF2α and XBP1, and consequently reducing CHOP mRNA expression, thereby protecting against CdCl_2_-induced renal injury [93]. Another animal study corroborates these findings, showing that quercetin significantly downregulates ERS markers, including GRP78, CHOP, PERK, IRE1α, and ATF6 in renal tissues, effectively ameliorating ERS in CKD [94]. Additionally, quercetin upregulates SLC7A11 expression, increases GSH and GPX4 levels, scavenges ROS, and decreases malondialdehyde (MDA) level, thereby mitigating ferroptosis-induced renal fibrosis [95].

Baicalin (BAI), a flavonoid obtained from the roots of *Scutellaria baicalensis* Georgi, regulates lipid metabolism disorders and demonstrates significant anti-inflammatory and antioxidant properties. Research shows that BAI activates the SIRT1/AMPK signaling pathway, alleviating lipid deposition in renal podocytes induced by high glucose conditions, thereby exerting renoprotective effects [96]. Additionally, BAI reduces urinary albumin-to-creatinine ratio (UACR) and urinary albumin excretion rate (UAER), while ameliorating pathological renal changes, including glomerular hypertrophy and mesangial matrix expansion. Its mechanisms involve activation of the Nrf2 signaling pathway, enhanced expression of antioxidant enzymes heme oxygenase-1 (HO-1) and NAD(P)H:quinone oxidoreductase 1 (NQO1), and suppression of NF-κB and MAPK signaling pathways, collectively mitigating chronic inflammation and oxidative stress [97]. Furthermore, considering the critical importance of strict glycemic control in CKD management, studies have demonstrated that the combination of baicalin with metformin produces a synergistic interaction, significantly enhancing the expression of antioxidant enzymes SOD and GPX, promoting ROS scavenge, and more effectively improving blood glucose and lipid profiles in diabetic rats compared to monotherapy, thereby providing protective effects against long-term hyperglycemia-induced renal injury [98]. However, the low oral bioavailability of BAI presents a limitation to clinical application. Zheng et al. demonstrated that a baicalin–lysozyme conjugate facilitates kidney-targeted delivery, increasing renal drug concentration, suppressing inflammation via NF-κB signaling, and inhibiting the TGF-β/Smad3 pathway to reduce extracellular matrix accumulation and renal fibrosis progression [99]. Notably, high-dose BAI treatment may abnormally activate the TGF-β/Smad pathway, potentially exacerbating renal injury and fibrosis [100].

Dihydromyricetin (DMY), extracted from *Ampelopsis grossedentata* (Hand. Mazz.) W. T. Wang, exhibits broad pharmacological activity against renal injury induced by insulin resistance and lipid accumulation. DMY enhances phosphorylation of insulin receptor substrate-1 (IRS1), restoring impaired insulin signaling pathways, improving insulin resistance, and reducing serum glucose and triglyceride concentrations, thereby ameliorating glucose and lipid metabolism disorders [101]. Moreover, DMY induces autophagy by inhibiting the PI3K/Akt/mTOR signaling pathway, reducing renal interstitial fibrosis and preserving renal function [102]. DMY also suppresses TGF-β1 expression and downregulates miR-34a, consequently promoting expression of Klotho, an endogenous inhibitor of renal fibrosis, thus improving renal fibrosis [103]. In vitro studies further confirm that DMY activates the Nrf2/HO-1 signaling pathway and upregulates NQO1 expression, inhibiting high glucose-induced extracellular matrix accumulation in human mesangial cells (HMCs) and reducing fibronectin (FN) expression, effectively attenuating renal fibrosis progression [104].

Chrysin, a flavonoid found abundantly in the propolis of *Populus przewalskii* Maxim., possesses notable anti-inflammatory and antioxidant properties. In gentamicin (GM)-induced renal injury models, chrysin upregulates the antioxidant system GSH/GPX activity and suppresses the NF-κB/kidney injury molecule-1 (KIM-1) signaling pathway, demonstrating significant anti-inflammatory and antioxidant capacities and alleviating renal pathological changes [105]. Additionally, chrysin enhances the Nrf2/HO-1-mediated antioxidant system, inhibits the RAGE/NLRP3 signaling pathway, attenuates CdCl_2_-induced renal inflammatory damage, suppresses pro-apoptotic proteins caspase-3/Bcl-2-associated X protein (Bax), and upregulates Bcl-2 expression, thereby reducing renal cell apoptosis and effectively delaying CKD progression [106].

Fisetin, a natural antioxidant abundant in fruits and vegetables, inhibits NF-κB-mediated inflammatory signaling and upregulates antioxidant enzymes, including GSH, NQO1, and SOD. It restores activities of mitochondrial respiratory chain enzymes, ameliorating mitochondrial dysfunction and alleviating renal inflammation and oxidative stress damage [107]. In CKD models, fisetin upregulates GSH and GPX4 levels, while suppressing overexpression of ACSL4 and cyclooxygenase-2 (COX-2), thereby inhibiting ferroptosis. Concurrently, it significantly reverses elevated expression of pro-inflammatory cytokines (IL-1β, IL-6, and TNF-α) and pro-fibrotic factors (α-SMA and fibronectin), effectively ameliorating tubular injury and tubulointerstitial fibrosis [108].

Isoliquiritigenin (ISL), isolated from the roots and rhizomes of *Glycyrrhiza uralensis* Fisch, suppresses expression of the innate immune receptor macrophage-inducible C-type lectin (Mincle), inhibits M1 macrophage polarization, upregulates SIRT1 expression, promotes NF-κB deacetylation, inhibits NLRP3 expression, and blocks secretion of inflammatory cytokines IL-1β, IL-6, and TNF-α, demonstrating significant anti-inflammatory effects in chronic kidney disease [109,110].

### 4.2. Polyphenols

Curcumin (CUR), extracted from the traditional medicinal plant *Curcuma longa* L., is one of the most widely used natural edible pigments globally. Research has revealed its broad pharmacological activities, including anti-inflammatory, antioxidant, and lipid-regulating effects. In CKD mice, CUR functions as a natural Nrf2 activator, scavenging free radicals and alleviating renal oxidative stress. Additionally, it suppresses NF-κB signaling and inhibits IL-1β release, thereby ameliorating inflammatory infiltration, tubular atrophy, and dilation. CUR also modulates apoptosis by downregulating Bcl-2-associated X protein (Bax) and upregulating Bcl-2 expression [111]. A double-blind randomized controlled trial demonstrated that curcumin supplementation downregulated NF-κB mRNA expression in peripheral blood and reduced plasma high-sensitivity C-reactive protein levels in hemodialysis patients, indicating potential anti-inflammatory benefits in CKD management [112]. Despite favorable patient compliance with oral administration, its clinical application is limited by its poor bioavailability. To address this limitation, a curcumin-phospholipid complex utilizing a novel phospholipid drug delivery system significantly enhances curcumin bioavailability, demonstrates a good safety profile, markedly reduces chronic inflammation in stage 3–4 CKD patients, and shows substantial therapeutic benefits [113]. Furthermore, given the multi-component, multi-target nature of natural medicines, their interactions warrant careful consideration. Studies indicate that curcumin and rhein exhibit significant synergistic effects in CKD treatment, with combination therapy reducing metabolic clearance, substantially increasing plasma drug concentrations, and more effectively improving renal fibrosis compared to monotherapy [114]. Notably, considering that conventional CKD management primarily focuses on strict control of blood pressure, glucose, and proteinuria, the potential integration of natural products with standard antihypertensive regimens represents a promising therapeutic approach. In this context, research has demonstrated that the combination of curcumin with amlodipine produces significantly enhanced vasodilatory effects compared to monotherapy, resulting in more effective blood pressure control and consequent renal protection [115].

Resveratrol (RSV), primarily obtained from *Reynoutria japonica* Houtt (Polygonaceae), promotes AMPK phosphorylation, subsequently activating the SIRT1/PGC1a signaling pathway and PPARA, while inhibiting the expression of SREBP-1—a key regulator of fatty acid synthesis—thereby regulating lipid metabolism disorders and ameliorating renal ectopic lipid deposition [116]. Additionally, resveratrol demonstrates significant anti-inflammatory and antioxidant capacities. Studies show that resveratrol upregulates renal Nrf2 expression; restores levels of antioxidant proteins, including HO-1, superoxide dismutase (SOD), and GPX; suppresses inflammatory mediators TNF-α and IL-6; repairs renal damage induced by inflammation and oxidative stress; and significantly reduces collagen deposition and fibrosis in renal tissues [117]. Furthermore, cotreatment with resveratrol and pioglitazone demonstrates superior efficacy in ameliorating metabolic disorders, significantly reducing hyperglycemia and improving insulin resistance compared to monotherapy, thereby providing enhanced protection against diabetes-induced renal injury [118].

Chlorogenic acid (CGA), a natural polyphenol, is found abundantly in *Ilex paraguariensis* A. St.-Hil. [119]. CGA inhibits neurogenic locus notch homolog protein 1 (Notch1) and signal transducer and activator of transcription (STAT3) protein expression, consequently attenuating SREBP-1c-mediated fatty acid synthesis while promoting CPT1-mediated fatty acid oxidation. This dual action reduces renal ectopic lipid deposition and suppresses the TGF-β1/Smad signaling pathway, thereby improving renal fibrosis [120]. Moreover, chlorogenic acid possesses potent anti-inflammatory and antioxidant properties. It inhibits Toll-like receptor 4 (TLR4)/NF-κB signal transduction and NLRP3 inflammasome activation, significantly mitigating renal inflammatory damage, while upregulating the Nrf2/HO-1 signaling pathway and enhancing SOD expression, thereby strengthening antioxidant capacity and effectively attenuating renal injury [121,122].

Carnosol, a natural compound extracted from *Rosmarinus officinalis* L., exhibits diverse biological activities and demonstrates therapeutic potential against various inflammatory diseases. Research confirms that carnosol provides significant protection against renal injury by downregulating pro-oxidant enzymes NOX and LOX, enhancing GSH levels and SOD activity, and exerting potent antioxidant effects to counteract oxidative stress. Simultaneously, it downregulates mRNA or protein expression of UPR signaling components—including GRP78, IRE1α, PERK, ATF4, ATF6, CHOP, XBP1s, and eIF2α—inhibiting endoplasmic reticulum stress, suppressing NF-κB cascade activation, blocking inflammatory cytokine release, and consequently improving renal function impairment [123].

### 4.3. Glycosides

Dioscin, extracted from *Dioscorea nipponica* Makino, has been investigated as a potential therapeutic agent for chronic kidney disease (CKD) due to its anti-inflammatory, antioxidant, and anti-fibrotic properties. Studies demonstrate that dioscin reduces phosphorylation of NF-κB both in vivo and in vitro, downregulates inflammatory factors including IL-1β, NLRP3, monocyte chemotactic protein 1 (MCP-1), IL-6, TNF-α, and IL-18, and diminishes inflammatory infiltration and collagen fiber deposition in renal tissues [124]. As key regulators of various biological processes, microRNAs are modulated by dioscin, which suppresses miR-34a expression and upregulates SIRT1, promoting nuclear translocation of Nrf2 and enhancing antioxidant gene transcription [125]. Furthermore, dioscin activates the Nrf2/HO-1 signaling pathway, enhances antioxidant capacity, scavenges ROS, upregulates GSH/GPX4 levels, and inhibits lipid peroxidation, thereby suppressing renal ferroptosis [126].

Astragaloside IV (AS-IV), one of the most bioactive compounds of *Astragalus membranaceus* (Fisch.) Bunge., attenuates NLRP3 inflammasome overexpression and reduces levels of IL-6, IL-1β, and TNF-α, thereby alleviating renal inflammatory injury [127]. Research indicates that AS-IV activates the Nrf2/HO-1/NQO1 pathway, promotes SOD expression, and eliminates ROS, significantly mitigating high glucose-induced oxidative stress in renal cells [128]. Additionally, AS-IV inhibits ERS-related proteins GRP78, PERK, ATF4, and CHOP, substantially reducing the Bax/Bcl-2 ratio and caspase-3 expression, thereby diminishing ERS-induced apoptosis in tubular epithelial cells and exerting beneficial effects against renal injury [129]. Furthermore, studies demonstrate that intravenous administration of AS-IV in combination with the RAAS inhibitor enalapril produces superior renoprotective effects compared to monotherapy, significantly improving urinary albumin excretion rate, providing better blood pressure control, and markedly reversing pathological changes, including glomerular basement membrane thickening, tubular cell proliferation, and tubular atrophy [130].

Ginsenoside Rb1 (Rb1), a bioactive component of *Panax ginseng* C. A. Mey., has been used for millennia in East Asian traditional medicine and demonstrates remarkable efficacy in treating various diseases. Rb1 inhibits NOX, thereby reducing renal oxidative stress, while downregulating TGF-β1, collagen I, and FN to suppress renal interstitial fibrosis in UUO rats [131]. It also downregulates GRP78 and eIF2α/CHOP signaling pathway to alleviate ERS and simultaneously suppresses the TGF-β1/Smad3 pathway, positioning it as a potential natural agent for treating renal fibrosis [132]. Clinical studies confirm that Rb1 supplementation upregulates GPX expression, reduces pro-inflammatory cytokines IL-6 and TNF-α, and effectively ameliorates oxidative stress and inflammation in early-stage CKD patients [133].

### 4.4. Polysaccharides

Astragalus polysaccharide, a key bioactive component of *Astragalus membranaceus* (Fisch.) Bunge., enhances AMPK activity and suppresses ACC expression, thereby improving glucose uptake and insulin sensitivity [134]. In high glucose-stimulated podocytes, TLR4 expression is significantly upregulated, while Astragalus polysaccharide treatment reverses this effect and inhibits subsequent NF-κB activation, thereby reducing IL-1β, IL-6, and TNF-α levels, suppressing inflammatory responses, and alleviating renal injury [135].

Ginseng polysaccharide, obtained from *Panax ginseng* C. A. Mey., significantly upregulates GSH and SOD expression, enhances renal antioxidant capacity, and eliminates ROS, thereby inhibiting oxidative stress-induced renal damage [136]. Additionally, it suppresses PERK/eIF2α/ATF4/CHOP signaling cascades, ameliorates ERS, and upregulates anti-apoptosis through the inhibition of Bax and upregulation of Bcl-2, collectively contributing to renal protection [137].

Refined fucose polysaccharide (RFP), extracted from the dry lateral roots of *Aconitum carmichaelii* Debeaux—commonly used for CKD—exhibits remarkable antioxidant capacity by effectively scavenging free radicals. It upregulates GSH/GPX4 and antioxidant enzyme SOD expression, ameliorates intracellular iron overload in renal cells, inhibits lipid peroxidation, significantly reduces lipid peroxide MDA and 4-hydroxynonenal (4-HNE), thereby effectively alleviating ferroptosis-induced renal injury [138].

### 4.5. Alkaloids

Nuciferine, extracted from the dried leaves of *Nelumbo nucifera* Gaertn., has demonstrated beneficial therapeutic effects against kidney diseases. Studies reveal that it activates AMPK, which subsequently downregulates FAS expression, suppresses fatty acid synthesis, and alleviates renal lipid deposition. Furthermore, AMPK activation promotes the Nrf2/HO-1 pathway while suppressing the TLR4/NF-κB pathway, thereby reducing oxidative stress and inflammation in CKD [139]. Nuciferine restores intracellular iron homeostasis by modulating FPN1 and TFR1, thereby attenuating renal iron overload. It also enhances the GSH/GPX4 antioxidant axis, upregulates SLC7A11 expression, and alleviates lipid peroxidation, further inhibiting ferroptosis in CKD, reducing fibronectin (FN) accumulation, and ameliorating renal fibrosis [140].

Berberine, isolated from *Coptis chinensis* Franch., promotes PGC1a expression, upregulates fatty acid oxidation-related enzymes, including CPT1 and PPARA, while activating the AMPK pathway and promoting ACC phosphorylation to suppress fatty acid synthesis. These processes collectively reduce lipid deposition in proximal tubular cells and ameliorate glomerulosclerosis and tubulointerstitial fibrosis [141]. Furthermore, berberine upregulates the Nrf2 signaling pathway, promotes Bcl-2 expression, downregulates NF-κB pathway, thereby enhancing antioxidant capacity and inhibiting renal apoptosis and inflammation [142].

Boldine, a principal alkaloid extracted from the bark and leaves of the traditional South American medicinal plant *Peumus boldus* Molina, has been demonstrated to attenuate renal inflammation and oxidative stress by reducing osteopontin (OPN) expression and macrophage infiltration, as well as by scavenging ROS. Boldine also inhibits TGF-β, significantly reducing the expression of α-SMA and collagen III, thus improving renal fibrosis [143]. Moreover, it promotes the expression of gap junction protein Connexin43 (Cx43), enhances intercellular communication among renal mesangial cells, reduces abnormally elevated membrane permeability, and helps maintain renal cellular homeostasis under high-glucose conditions, thereby curbing the localized amplification of inflammation [144].

### 4.6. Quinones

Emodin, a bioactive anthraquinone extracted from *Rheum palmatum* L., exerts multifaceted renoprotective effects through several mechanisms. It upregulates the AMPK/mTOR signaling pathway to promote renal autophagy and preserve renal function [145]. Furthermore, emodin enhances antioxidant defense via Nrf2 activation and suppresses inflammation by inhibiting NF-κB signaling [146]. Studies show that emodin can also ameliorate high glucose-induced ERS by inhibiting PERK/eIF2α/ATF4/CHOP pathways, thereby reducing apoptosis and attenuating renal injury [147]. However, findings from the National Toxicology Program indicate a significant dose-dependent safety concern, with mice exposed to high concentrations of emodin showing markedly increased incidence and severity of renal tubule injury and nephropathy [148].

Rhein, isolated from *Rheum palmatum* L., ameliorates renal fibrosis by inhibiting STAT3 phosphorylation and suppressing the expression of collagen I and α-smooth muscle actin (α-SMA). It also attenuates renal cell apoptosis by upregulating Bcl-2 and downregulating Bax [149]. Additionally, rhein suppresses TFR1 expression, thereby ameliorating intracellular iron overload. Concurrently, it inhibits ACSL4 activity to reduce lipid peroxidation substrate generation and upregulates GSH/GPX4 activity to enhance ROS scavenging, ultimately blocking the onset of ferroptosis [150]. However, caution must be exercised regarding the dose-dependent safety issues associated with rhein. Studies have demonstrated that long-term administration of rhein can significantly reduce SOD and GSH levels, promote the expression of TGF-β1 and TNF-α, increase blood urea nitrogen and serum creatinine levels, and exacerbate pathological renal damage, indicating its potential nephrotoxicity [151].

Tanshinone IIA, extracted from *Salvia miltiorrhiza* Bunge, exerts renoprotective effects through multi-pathway regulation. It attenuates renal inflammation and fibrosis by inhibiting NF-κB and TGF-β/Smad signaling pathways [152]. Furthermore, it also alleviates oxidative stress and pyroptosis through scavenging ROS, downregulating NLRP3 expression, and suppressing caspase-1-mediated GSDMD cleavage [153]. Additionally, it mitigates endoplasmic reticulum stress in CKD by inhibiting PERK/eIF2α/ATF4/CHOP and reducing GRP78 expression, thereby preserving renal function [154].

### 4.7. Terpenoids

Oleanolic acid (OA), a natural pentacyclic triterpenoid from *Olea europaea* L., exhibits broad pharmacological activities. By activating the AMPK/PGC1a pathway to enhance fatty acid metabolism and alleviate renal lipid deposition, while simultaneously suppressing the TLR4/NF-κB axis to inhibit pro-inflammatory mediator production and mitigate inflammatory injury, OA confers renoprotective effects [155]. OA also attenuates oxidative stress by upregulating SOD expression and enhancing ROS scavenging, while concurrently suppressing ERS through inhibition of the PERK/eIF2α/CHOP pathway. Furthermore, OA downregulates TGF-β/Smad signaling to mitigate renal fibrosis and reduces apoptosis by upregulating Bcl-2 expression, collectively attenuating renal injury [156]. Additionally, in diabetic rat models, OA demonstrates synergistic effects with insulin by enhancing insulin signaling pathway activation, promoting glycogen synthase expression, reducing glycogen levels, and consequently ameliorating hyperglycemia-induced organ damage [157]. However, the clinical translation of OA is limited by the poor bioavailability and lack of targeting specificity of conventional oral formulations. To address these challenges, Chen et al. developed a biomimetic drug delivery system consisting of neutrophil membrane-coated liposomes loaded with OA. This innovative platform significantly improves renal targeting, enhances antioxidant and anti-inflammatory efficacy, and promotes renal functional recovery [158].

Withaferin A, a bioactive compound isolated from the traditional Indian medicinal plant *Withania somnifera* Dunal, demonstrates significant renoprotective effects in CKD. It effectively attenuates renal inflammation by suppressing NF-κB phosphorylation and IL-1β release, while concurrently inhibiting the expression of pro-fibrotic mediators, including TGF-β and fibronectin. Furthermore, withaferin A ameliorates endoplasmic reticulum stress through downregulation of the eIF2α/ATF4/CHOP axis and GRP78 overexpression, collectively contributing to its renal protective activity [159].

Ginkgolide B (GB), a bioactive terpenoid from the leaves of *Ginkgo biloba* L., alleviates renal ferroptosis and oxidative stress by modulating iron homeostasis and enhancing antioxidant capacity. It upregulates ferritin heavy chain 1 (FTH1) and inhibits TFR1 activity, thereby maintaining intracellular iron homeostasis. Concurrently, GB activates GPX4 and promotes ROS scavenging, effectively ameliorating renal oxidative stress and ferroptosis [160].

### 4.8. Others

Sulforaphane (SFN), an isothiocyanate compound extracted from *Brassica oleracea* var. Italica Plenck, demonstrates a renoprotective effect through the regulation of lipid metabolism, mitochondrial function, and oxidative stress. SFN activates AMPK, ameliorating lipid metabolic disorders and attenuating renal lipotoxic injury [161]. It also downregulates the renal scavenger receptor CD36, suppresses SREBP-1 and FAS to inhibit fatty acid synthesis, and effectively reduces renal lipid deposition. Concurrently, SFN upregulates PGC1a and Nrf1, enhances mitochondrial biogenesis, thereby alleviating kidney injury [162]. In vitro studies have further confirmed that sulforaphane (SFN) upregulates Nrf2 activity, promotes NQO1 expression, and scavenges reactive oxygen species (ROS). Concurrently, SFN elevates glutamate-cysteine ligase (GCL) expression, enhances glutathione (GSH) biosynthesis, and thereby synergistically counteracts cisplatin-induced oxidative stress, ultimately alleviating renal injury [163]. Furthermore, a clinical study involving non-dialysis CKD patients revealed that daily supplementation with 400 μg SFN for one month significantly increased Nrf2 and NQO1 expression, enhanced antioxidant capacity, and mitigated oxidative stress-mediated renal injury [164].

Brazilian green propolis (BGP), a natural product extracted from resin collected by bees from *Baccharis dracunculifolia* DC., is rich in various bioactive components. Clinical studies confirm that supplementation with BGP extract alleviates inflammation and oxidative stress in dialysis patients with CKD, through suppression of NF-κB, reduction in pro-inflammatory cytokines including TNF-α and IL-1β, and activation of the Nrf2 pathway [165,166].

*Cordyceps sinensis* (Berk.) Sacc. (CS), a fungal–larval complex used traditionally in renal diseases, regulates renal lipid metabolism disorders by activating PPARA and inhibiting FAS [167]. It also attenuates renal fibrosis through suppression of the TGF-β1/Smad signaling pathway and inhibition of renal epithelial–mesenchymal transition (EMT) [168]. Furthermore, in chronic allograft nephropathy patients, CS combined with immunosuppressants significantly reduces the incidence of complications and is beneficial for improving renal function [169].

**Table 1:** Potential bioactive components for the treatment of CKD.

Categories	Natural Products	Herb	Model	Dose	Effects	Mechanisms	Ref.
Flavonoids	Quercetin	Various herbs	Cd-induced SD rats	50, 100 mg/kg	AMPK↑, PPARA↑, CPT1↑, SREBP-1↓, TG↓; NF-κB↓, TNF-α↓, TGF-β1↓; SIRT1↑, eIF2α↓, XBP1↓, CHOP↓, GRP78↓, PERK↓, IRE1α↓, ATF6↓; SLC7A11↓, GSH↑, GPX4↑, ROS↓, MDA↓	Improve metabolic dysregulation, anti-inflammation, inhibit endoplasmic reticulum stress, and inhibit ferroptosis	[91]
			HG-induced SV40 MES 13 cells	5, 10, 50 μg/L			[92]
			Cd-induced Wistar rats	50 mg/kg/d			[93]
			Adenine andPotassium oxonate-induced SD rats	50, 100 mg/kg			[94]
			FA/IRI-induced C57BL/6J mice	25 mg/kg			[95]
			RSL3/erastin-induced HK-2/NRK-52E cells	20 μM			
	Baicalin	*Scutellaria baicalensis* Georgi	db/db mice	100 mg/kg	SIRT1↑, AMPK↑; Nrf2↑, HO-1↑, NQO1↑; NF-κB↓, MAPK↓, TGF-β↓, Smad3↓	Improve metabolic dysregulation, anti-inflammation, antioxidative stress, and reduce fibrosis	[96]
			HG-induced MPC-5 cells	3, 6, 12 μM			
			db/db mice	400 mg/kg			[97]
			STZ-induced SD rats	160 mg/kg			[99]
	Dihydromyricetin	*Ampelopsis grossedentata* (Hand. Mazz.) W. T. Wang	HFD-induced db/db mice	500, 1000 mg/kg	IRS1↑, PI3K↓, Akt↓, mTOR↓, TGF-β1↓, Nrf2↑, HO-1↑, NQO1	Improve metabolic dysregulation, promote autophagy, and antioxidative stress	[101]
			STZ-induced SD rats	100 mg/kg			[102]
			HG induced NRK-52E/HEK293 cells	1 μM			
			UUO C57BL/6J mice	500 mg/kg			[103]
			TGF-β1-induced HK-2 cells	50, 100, 200 μM			
			HG-induced HMC cells	10, 20 μM			[104]
	Chrysin	*Populus przewalskii* Maxim.	GM-induced albino rats	100 mg/kg	GSH↑, GPX↑, Nrf2↑, HO-1↑, NF-κB↓, RAGE↓, NLRP3↓, caspase-3↓, Bax↓, Bcl-2↑	Antioxidative stress, anti-inflammation, and reduce apoptosis	[105]
			Cd-induced Wistar albino rats	25, 50 mg/kg			[106]
	Fisetin	Various herbs	CIS-induced SD rats	0.625, 1.25 mg/kg	NF-κB↓, GSH↑, NQO1↑, SOD↑, GPX4↑, ACSL4↓, COX-2↓, IL-1β↓, IL-6↓, TNF-α↓, α-SMA↓, FN↓	Antioxidative stress, anti-inflammation, inhibit ferroptosis, reduce fibrosis	[107]
			Adenine/UUO induced C57BL/6J mice	50, 100 mg/kg			[108]
			Adenine/TGF-β1 induced TCMK-1 cells	20 μM			
	Isoliquiritigenin	*Glycyrrhiza uralensis* Fisch	UUO induced C57BL/6J mice	7.5, 30 mg/kg	SIRT1↑, NF-κB↓, NLRP3↓, IL-1β↓, IL-6↓, TNF-α↓	Anti-inflammation	[109]
			STZ-induced SD rats	20 mg/kg			[110]
Polyphenols	Curcumin	*Curcuma longa* L.	GM-induced albino rats	200 mg/kg	Nrf2↑, ROS↓, NF-κB↓, IL-1β↓, Bax↓, Bcl-2↑	Antioxidative stress, anti-inflammation, and reducing apoptosis	[111]
			Hemodialysis CKD patients	2.5 g (95% purity) post-dialysis			[112]
			CKD 3–4 patients	500 mg twice a day			[113]
	Resveratrol	*Reynoutria japonica* Houtt	C57BLKS/J db/db mice	20 mg/kg	SIRT1↑, PGC1a↑, PPARA↑, SREBP-1↓, Nrf2↑, HO-1↑, SOD↑, GPX↑, TNF-α↓, IL-6↓	Improve metabolic dysregulation, antioxidative stress, and anti-inflammation	[116]
			HFD-induced Wistar rats	100 mg/kg			[117]
	Chlorogenic acid	*Ilex paraguariensis* A. St.-Hil.	HFD+STZ-induced C57BL/6J mice	50 mg/kg	Notch1↓, STAT3↓, SREBP-1c↓, CPT1↑, TGF-β1↓, Smad↓, TLR4↓, NF-κB↓, NLRP3↓, Nrf2↑, HO-1↑, SOD↑	Improve metabolic dysregulation, antioxidative stress, anti-inflammation, and reduce fibrosis	[120]
			HG+PA induced HK-2 cells	20, 40, 80 μM			
			IRI-induced Swiss mice	3.5, 7, 14 mg/kg			[121]
			HFD+STZ-induced Wistar rats	10 mg/kg			[122]
			HG-induced HK-2 cells	20, 50, 100 μM			
	Carnosol	*Rosmarinus officinalis* L.	UUO-induced C57BL/6J mice	50 mg/kg	NOX↓, LOX↓, GSH↑, SOD↑, GRP78↓, IRE1α↓, PERK↓, ATF4↓, ATF6↓, CHOP↓, XBP1↓, eIF2α↓, NF-κB↓	Antioxidative stress, anti-inflammation, and inhibit endoplasmic reticulum stress	[123]
Glycosides	Dioscin	*Dioscorea nipponica* Makino	UUO-induced C57BL/6J mice	50, 100 mg/kg	NF-κB↓, IL-1β↓, NLRP3↓, MCP-1↓, IL-6↓, TNF-α↓, IL-18↓, SIRT1↑, Nrf2↑, HO-1↑, ROS↓, GSH↑, GPX4↑	Antioxidative stress, anti-inflammation, inhibit ferroptosis	[124]
			TGF-β1-induced HK-2 cells	3.125, 6.25, 12.5 μM			
			CIS-induced HK-2/NRK-52E cells	50,100, and 200 ng/mL			[125]
			CIS-induced Wistar rats/C57BL/6J mice	10, 20, 40 mg/kg; 10, 30, 60 mg/kg			
			CIS-induced Wistar rats	60 mg/kg			[126]
	Astragaloside IV	*Astragalus membranaceus* (Fisch.) Bunge	db/db mice	40 mg/kg	NLRP3↓, IL-6↓, IL-1β↓, TNF-α↓, Nrf2↑, HO-1↑, NQO1↑, SOD↑, ROS↓, GRP78↓, PERK↓, ATF4↓, CHOP↓, Bax↓, Bcl-2↑, and caspase-3↓	Antioxidative stress, anti-inflammation, inhibit endoplasmic reticulum stress, and reduce apoptosis	[127]
			HG-induced MPC cells	10, 20, 40 μM			
			HG-induced HK-2 cells	10, 20, 40 μM			[128]
			HFD+STZ-induced SD rats	20, 40, 80 mg/kg			[129]
	Ginsenoside Rb1	*Panax ginseng* C. A. Mey.	UUO SD rats	50 mg/kg	NOX↓, TGF-β1↓, Smad3↓, collagen I↓, FN↓, GRP78↓, eIF2α↓, CHOP↓, GPX↑, IL-6↓, TNF-α↓	Antioxidative stress, anti-inflammation, inhibit endoplasmic reticulum stress, reduce fibrosis	[131]
			Bavachin-induced HK-2 cells	40 μM			[132]
			CKD 2–3 patients	500 mg/d			[133]
Polysaccharides	Astragalus polysaccharide	*Astragalus membranaceus* (Fisch.) Bunge.	HFD+STZ-induced SD rats	700 mg/kg	AMPK↑, ACC↓, TLR4↓, NF-κB↓, IL-1β↓, IL-6↓, TNF-α↓	Improve metabolic dysregulation, anti-inflammation	[134]
			STZ-induced SD rats	200, 400, 800 mg/kg			[135]
	Ginseng polysaccharide	*Panax ginseng* C. A. Mey.	Cr-induced ICR mice	25, 50, 100, 200, 400 mg/kg	GSH↑, SOD↑, ROS↓, PERK↓, eIF2α↓, ATF4↓, CHOP↓, Bax↓, Bcl-2↑	Antioxidative stress, inhibit endoplasmic reticulum stress, and reduce apoptosis	[136]
			CIS-induced ICR mice	200, 400 mg/kg			[137]
	Refined fucose polysaccharide	*Aconitum carmichaelii* Debeaux	CIS-induced Kunming mice	200, 400, 800 mg/kg	GSH↑, GPX4↑, SOD↑, MDA↓, 4-HNE↓	Antioxidative stress, and inhibit ferroptosis	[138]
Alkaloids	Nuciferine	*Nelumbo nucifera* Gaertn.	PA-induced HK-2 cells	10, 40 μM	AMPK↑, FAS↓, Nrf2↑, HO-1↑, TLR4↓, NF-κB↓, FPN1↑, TFR1↓, GSH↑, GPX4↑, SLC7A11↑, FN↓	Improve metabolic dysregulation, antioxidative stress, anti-inflammation, and inhibit ferroptosis	[139]
			FA-induced C57BL/6 mice	30 mg/kg			[140]
			RSL3/erastin/FIN56 induced HK-2/HEK293T cells	2.5, 5, 10, 20, 40 μM			
	Berberine	*Coptis chinensis* Franch.	C57BLKS/J db/db mice	200 mg/kg	PGC1a↑, CPT1↑, PPARA↑, ACC↓, Nrf2↑, Bcl-2↑, NF-κB↓	Improve metabolic dysregulation, anti-inflammation, antioxidative stress, and reduce apoptosis	[141]
			PA-induced MPC-5 cells	0.4 μM			
			Methotrexate induced male albino rats	200 mg/kg			[142]
	Boldine	*Peumus boldus* Molina	Two-Kidney, One-Clip induced SD rats	50 mg/kg	OPN↓, ROS↓, TGF-β↓, α-SMA↓, collagen III, ↓, Cx43↑	Antioxidative stress, anti-inflammation, and reducing fibrosis	[143]
			STZ-induced SD rats	50 mg/kg			[144]
Quinones	Emodin	*Rheum palmatum* L.	STZ-induced SD rats	20, 40 mg/kg	AMPK↑, mTOR↓, Nrf2↑, NF-κB↓, PERK↓, eIF2α↓, ATF4↓, CHOP↓	Promote autophagy, antioxidative stress, anti-inflammation, and inhibit endoplasmic reticulum stress	[145]
			adenine-induced SD rats	100 mg/kg			[146]
			KK-Ay mice	40, 80 mg/kg			[147]
			HG-induced MCP-5 cells	20, 40 μM			
	Rhein	*Rheum palmatum* L.	UUO SD rats	150 mg/kg	STAT3↓, α-SMA↓, Bax↓, Bcl-2↑, TFR1↓, ACSL4↓, GSH↑, GPX4↑, ROS↓	Antioxidative stress, inhibiting ferroptosis, and reducing apoptosis and fibrosis	[149]
			STZ-induced C57BL/6J mice	150 mg/kg			[150]
			HG-induced MPC-5 cells	25 μg/ml			
	Tanshinone IIA	*Salvia miltiorrhiza* Bunge	5/6 nephrectomy induced SD rats	10 mg/kg	NF-κB↓, TGF-β↓, Smad↓, ROS↓, caspase-1↓, GSDMD↓, PERK↓, eIF2α↓, ATF4↓, CHOP↓, GRP78↓	Antioxidative stress, anti-inflammation, inhibit endoplasmic reticulum stress, reduce pyroptosis	[152]
			db/db mice	10 mg/kg			[153]
			HG-induced HRGEC cells	20 μg/ml			
			STZ-induced SD rats	2, 4, 8 mg/kg			[154]
Terpenoids	Oleanolic acid	*Olea europaea* L.	HFD+STZ induced SD rats	50, 100 mg/kg	AMPK↑, PGC1a↑, TLR4↓, NF-κB↓, SOD↑, ROS↓, PERK↓, eIF2α↓, CHOP↓, TGF-β↓, Smad↓, Bcl-2↑	Improve metabolic dysregulation, anti-inflammation, antioxidative stress, inhibit endoplasmic reticulum stress, and reduce apoptosis	[155]
			OLETF rats	5 μM			[156]
			H_2_O_2_-induced NRK-52E cells	8 μM			[158]
	Withaferin A	*Withania somnifera Dunal*	UUO-induced C57BL/6J mice	3 mg/kg/d	NF-κB↓, IL-1β↓, TGF-β↓, FN↓, eIF2α↓, ATF4↓, CHOP↓, GRP78↓	Anti-inflammation, inhibit endoplasmic reticulum stress, reduce fibrosis	[159]
	Ginkgolide B	*Ginkgo biloba* L.	C57BL/KsJ db/db mice	200 mg/kg	FTH1↑, TFR1↓, GPX4↑, ROS↓	Antioxidative stress and inhibit ferroptosis	[160]
			PA+HG-induced MCP-5 cells	20, 40, 80 μM			
Others	Sulforaphane	*Brassica oleracea var. Italica Plenck*	HFD+STZ induced C57BL/6J mice	0.5 mg/kg	AMPK↑, CD36↓, SREBP-1↓, FAS↓, PGC1a↑, Nrf1↑, Nrf2↑, NQO1↑, ROS↓, GCL↑, GSH↑	Improve metabolic dysregulation, antioxidative stress	[161]
			UUO Wistar rats	1 mg/kg			[162]
			CIS-induced LLC-PK1 cells	1, 3, 5 μM			[163]
			CIS-induced Wistar rats	500 μg/g			
			Non-dialysis CKD patients	400 μg/d			[164]
	Brazilian green propolis	*Baccharis dracunculifolia DC.*	Peritoneal dialysis CKD patients	400 mg/d	NF-κB↓, TNF-α↓, IL-1β↓, Nrf2↑	Antioxidative stress, anti-inflammation	[165]
			Hemodialysis CKD patients	200 mg/d			[166]
	*Cordyceps sinensis* extract	*Cordyceps sinensis* (Berk.) Sacc.	HFD+STZ-induced SD rats	1.2 g/kg	PPARA↑, FAS↓, TGF-β1↓, Smad↓, EMT↓	Improve metabolic dysregulation, reduce fibrosis	[167]
			5/6 nephrectomy induced SD rats	2 g/kg			[168]
			Chronic allograft nephropathy patients	2.0 g/d			[169]

↑ (Upward arrow): represents upregulation. ↓ (Downward arrow): represents downregulation.

## 5. Conclusions

Chronic kidney disease (CKD) is characterized by a complex pathological network involving interconnected mechanisms such as metabolic disturbances, chronic inflammation, oxidative stress, endoplasmic reticulum stress (ERS), and ferroptosis. These processes engage multiple signaling molecules and pathways, collectively driving renal injury. Rather than stemming from a single etiology, CKD progression is orchestrated by a vicious cycle of these interconnected pathological events. Natural products have recently emerged as promising candidates for therapeutic intervention, largely owing to their multi-target, multi-pathway synergistic properties. Extensive preclinical evidence indicates that various natural compounds can effectively modulate core pathological processes in CKD, thereby producing synergistic benefits in attenuating renal injury, thereby complementing conventional therapies aimed at controlling blood pressure, glucose, and proteinuria.

While the pleiotropic nature of natural products offers therapeutic advantages for multifaceted conditions like CKD, current evidence predominantly derives from preclinical studies. Their clinical translation is hindered by the absence of large-scale, multicenter, randomized controlled trials. Additionally, some natural products present potential toxicities—for instance, high-dose baicalin has been reported to abnormally activate the TGF-β/Smad pathway and aggravate renal fibrosis. Notably, while the potential toxicities of certain natural products have been well-documented in other disease models, direct evidence within the specific physiological condition of CKD is still lacking. Future studies should prioritize the systematic evaluation of their safety profiles under CKD conditions. Beyond safety concerns, significant pharmacokinetic obstacles impede translation. Many natural compounds exhibit poor oral bioavailability, extensive first-pass metabolism, and rapid systemic clearance, resulting in subtherapeutic concentrations at the renal level. Furthermore, the substantial heterogeneity in quality control, extraction methodologies, dosage protocols, and efficacy endpoints across studies creates reproducibility issues and prevents the establishment of standardized, universally applicable treatment regimens. This combination of insufficient clinical validation, uncertain safety profiles, suboptimal pharmacokinetics, and methodological variability collectively hinders the development of evidence-based, standardized natural product therapies for CKD.

Notwithstanding these limitations, the therapeutic potential of natural products in retarding CKD progression remains considerable. Future research should prioritize elucidating the molecular mechanisms of natural products in CKD. A particularly promising direction lies in exploring their synergistic effects with conventional CKD treatment regimens. Investigating these synergies could unlock strategies to enhance therapeutic efficacy, reduce dosages of conventional drugs—thereby minimizing their side effects—and potentially overcome drug resistance, ultimately leading to more comprehensive and personalized treatment strategies for CKD. To overcome pharmacokinetic challenges such as poor oral bioavailability and rapid clearance, innovative delivery systems, such as curcumin-phospholipid complexes, renal-targeted baicalin-lysozyme conjugates, and neutrophil membrane-coated oleanolic acid liposomes, should be developed to enhance both bioavailability and kidney-specific targeting. Concurrently, rigorously designed randomized controlled trials are imperative to systematically evaluate clinical efficacy, long-term safety, and potential side effects of natural products in CKD, thereby providing robust evidence for clinical translation.

In summary, natural products show considerable therapeutic potential in CKD management. With advances in mechanistic elucidation, optimized delivery systems, strengthened clinical evidence, and standardized preparation protocols, natural products are positioned to become integral components of comprehensive CKD treatment strategies, thereby providing novel therapeutic avenues for patients worldwide.

## Data Availability

No new data were created or analyzed in this study. Data sharing is not applicable.

## Abbreviations

The following abbreviations are used in this manuscript:

ACC	Acetyl-CoA carboxylase
ACSL4	Acyl-CoA synthetase long chain family member 4
ALB	Albumin
AMPK	AMP-activated protein kinase
AngII	Angiotensin II
ATF4	Activating transcription factor 4
ATF6	Activating transcription factor 6
α-SMA	Alpha-smooth muscle actin
Bax	Bcl-2-associated X protein
Bcl-2	B-cell lymphoma-2
BIM	Bcl-2 interacting mediator of cell death
β2-MG	β2-microglobulin
CHOP	C/EBP homologous protein
ChREBP	Carbohydrate response element-binding protein
CKD	Chronic kidney disease
COX-2	Cyclooxygenase-2
CPT1	Carnitine palmitoyltransferase-1
Cx43	Connexin43
ECM	Extracellular matrix
eIF2α	Eukaryotic initiation factor 2α
EMT	Epithelial-mesenchymal transition
ERS	Endoplasmic reticulum stress
ESRD	End-stage renal disease
ESRRA	Estrogen-related receptor alpha
FAO	Fatty acid oxidation
FAS	Fatty acid synthase
FFAs	Free fatty acids
FN	Fibronectin
FPN	Ferroportin
FTH1	Ferritin heavy chain 1
GCL	Glutamate-cysteine ligase
GM	Gentamicin
GPX4	Glutathione peroxidase 4
GRP78	Glucose-regulated protein 78
GSH	Glutathione
GSDMD	Gasdermin D
HMCs	Human mesangial cells
HO-1	Heme oxygenase-1
IL-18	Interleukin-18
IL-1β	Interleukin-1β
IL-6	Interleukin-6
IRE1α	Inositol-requiring enzyme 1 alpha
IRS1	Insulin receptor substrate-1
JNK	C-Jun N-terminal kinase
KIM-1	Kidney injury molecule-1
LIP	Labile iron pool
LOX	Lipoxygenase
LPCAT3	Lysophosphatidylcholine acyltransferase 3
MCP-1	Monocyte chemotactic protein 1
MDA	Malondialdehyde
Mincle	Macrophage-inducible C-type lectin
NF-κB	Nuclear factor kappa-B
Notch1	Neurogenic locus notch homolog protein 1
NOX	NADPH oxidase
NQO1	NAD(P)H:quinone oxidoreductase 1
Nrf2	Nuclear factor erythroid 2-related factor 2
OPN	Osteopontin
OXPHOS	Oxidative phosphorylation
PERK	Protein kinase RNA-like endoplasmic reticulum kinase
PGC1a	Peroxisome proliferator-activated receptor gamma coactivator 1-alpha
PPARA	Peroxisome proliferator-activated receptor alpha
POR	Cytochrome P450 oxidoreductase
PRR	Pro-renin receptor
PUFA	Polyunsaturated fatty acid
PUFA-PE	Polyunsaturated fatty acid-phosphatidyl ethanolamine
RAASi	Renin–angiotensin–aldosterone system inhibitor
RAS	Renin–angiotensin system
ROS	Reactive oxygen species
SGLT2i	Sodium-glucose cotransporter-2 inhibitor
SIRT1	Sirtuin-1
SOD	Superoxide dismutase
SREBP-1c	Sterol regulatory element-binding protein-1c
STAT3	Signal transducer and activator of transcription 3
TGF-β	Transforming growth factor-beta
TFR1	Transferrin receptor 1
TLR4	Toll-like receptor 4
TNF-α	Tumor necrosis factor-alpha
TRAF2	TNF receptor-associated factor 2
UACR	Urinary albumin-to-creatinine ratio
UAER	Urinary albumin excretion rate
UPR	Unfolded protein response
XBP1	X-box binding protein 1
4-HNE	4-hydroxynonenal
